# Yabi: An online research environment for grid, high performance and cloud computing

**DOI:** 10.1186/1751-0473-7-1

**Published:** 2012-02-15

**Authors:** Adam A Hunter, Andrew B Macgregor, Tamas O Szabo, Crispin A Wellington, Matthew I Bellgard

**Affiliations:** 1 Centre for Comparative Genomics, Murdoch, Western Australia, 6150

**Keywords:** Bioinformatics, workflows, Internet, high performance computing

## Abstract

**Background:**

There is a significant demand for creating pipelines or workflows in the life science discipline that chain a number of discrete compute and data intensive analysis tasks into sophisticated analysis procedures. This need has led to the development of general as well as domain-specific workflow environments that are either complex desktop applications or Internet-based applications. Complexities can arise when configuring these applications in heterogeneous compute and storage environments if the execution and data access models are not designed appropriately. These complexities manifest themselves through limited access to available HPC resources, significant overhead required to configure tools and inability for users to simply manage files across heterogenous HPC storage infrastructure.

**Results:**

In this paper, we describe the architecture of a software system that is adaptable to a range of both pluggable execution and data backends in an open source implementation called Yabi. Enabling seamless and transparent access to heterogenous HPC environments at its core, Yabi then provides an analysis workflow environment that can create and reuse workflows as well as manage large amounts of both raw and processed data in a secure and flexible way across geographically distributed compute resources. Yabi can be used via a web-based environment to drag-and-drop tools to create sophisticated workflows. Yabi can also be accessed through the Yabi command line which is designed for users that are more comfortable with writing scripts or for enabling external workflow environments to leverage the features in Yabi. Configuring tools can be a significant overhead in workflow environments. Yabi greatly simplifies this task by enabling system administrators to configure as well as manage running tools via a web-based environment and without the need to write or edit software programs or scripts. In this paper, we highlight Yabi's capabilities through a range of bioinformatics use cases that arise from large-scale biomedical data analysis.

**Conclusion:**

The Yabi system encapsulates considered design of both execution and data models, while abstracting technical details away from users who are not skilled in HPC and providing an intuitive drag-and-drop scalable web-based workflow environment where the same tools can also be accessed via a command line. Yabi is currently in use and deployed at multiple institutions and is available at http://ccg.murdoch.edu.au/yabi.

## Background

Chaining a number of analysis tools together to form domain-specific analysis pipelines or workflows is essential in many scientific disciplines [1-3]. For some scientists access to a command line login is all that is required for them to write custom scripts and programs to link these tasks. For instance, workflows can be implemented in programming languages such as Perl (http://www.perl.org/), Python (http://www.python.org/) or Java (http://java.sun.com/), utilising extensive libraries such as Bioperl [4] and Biojava [5] and BioPython (http://biopython.org). More recently tools and data can be accessed via web services [6,7]. However, constructing analysis workflows in this manner requires a level of programming proficiency that typically presents a barrier to many scientists [8-10]. In addition, the amount of data and the compute intensive nature of the tasks demand the need to run these tasks on large-scale high performance computing (HPC) infrastructure. Unfortunately, existing tools for interacting with the Grid or HPC resources such as Globus [11] and super computer scheduling systems are often too "low level" and require computing knowledge that is also outside the expertise of many scientists.

Simple, transparent access to High Performance Computing (HPC) and Grid resources for non-technical users is a significant challenge that must be overcome to facilitate widespread adoption of large-scale compute and data resources [8-10]. In trying to address this, significant work has been undertaken to develop workflow environments that attempt to alleviate the need of having scientists write their own scripts or programs. These are either desktop applications or more recently Internet-based applications [12-16]. Two examples are now provided.

Taverna is a powerful desktop workflow management system [12,13]. However, to fully exploit the power of this system requires a high level of programming competence. Firstly, users are provided a desktop application that offers extreme flexibility and configurability but can be complex and difficult to uptake by users that simply want to quickly chain a core set of tools together. Secondly, the application has an execution model that either runs executables on the user's local machine or submits data to web services for execution. A typical Taverna installation does not have a data model other than to manage local files. Thirdly, scientists must incorporate error handling for any tools they wish to incorporate in each workflow. For example, the error messages from a failed web service call in a given workflow can be quite daunting to non-HPC trained scientists.

In other words, scientists using Taverna must manage the complexity of the workflow, the tools to be administered, and the error handling of the tools. The end users of the system cannot hide these exposed details of the compute infrastructure or manage the tools in a simplified way. For many users this is sufficient, but for many other users who desire an intuitive browser-based workflow environment, systems such as Taverna may not be appropriate.

Galaxy is a rich domain-specific Internet-based environment tailored to bioinformatics genomics data anlaysis [14]. The interface is considerably simplified for scientists to use compared to Taverna. In Galaxy, the role of maintaining tools is separated from the user, although it requires the skills of a software developer to add new tools to the system. For instance, XML scripts are required to be edited for both tool configuration and to notify Galaxy of the tool's existence [17]. While this is manageable in some instances, configuring a large number of tools for a range of different user groups would demand significant overhead. While Galaxy is specifically designed for genomics analysis, more recently Galaxy is being extended to introduce proteomics data types [18].

As highlighted by the previous two examples, there exists a need for scientific workflow environments that have the following necessary features: Internet-based and intuitive to use; tool management abstracted from users; defined process for expanding the list of available tools and custom scripts that do not require software development expertise; scientists are not tied to the workflow environment to access data and results (prevent data lock in); provide comprehensive configuration for execution and data access models to leverage existing compute and data infrastructure.

In this paper, we introduce Yabi, an Internet-based interface to a workflow engine that solves the problem of workflow deployment across disparate legacy HPC resources. Yabi abstracts the complexity involved in accessing multiple HPC resources and data stores from the scientific researcher. In this way Yabi enables researchers access to HPC power without requiring specialised computing knowledge.

## Implementation

### 1 Overview

**Figure 1:**
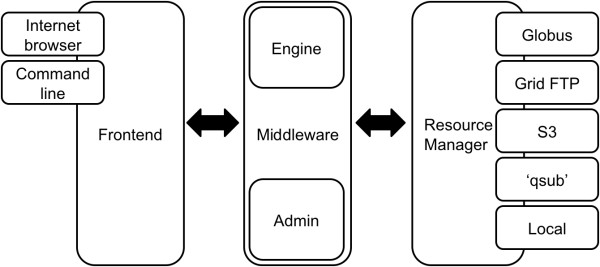
**Yabi Architecture**. The YABI architecture outlining the key components including Frontend Application, Middleware Appliance and the Resource Manager.

Yabi uses a three-tier architecture to enable flexibility and reuse [19]. The first layer is the frontend web application that provides the main user interface; the second layer is the middleware that is responsible for process management, tool configuration, analysis audit trails [1,20] and user management; the third layer is the Resource Manager that exposes data and compute resources to the middleware. The core components are shown in Figure 1.

Yabi simplifies the visual representation of the workflow to the scientist by highlighting the files and parameters that are relevant while hiding from the scientist the computational structures such as branching, looping, conditionals, dependency resolution and fault recovery. Yabi shifts the complexities of managing the infrastructure away from the end user and onto the system administrator responsible for managing the HPC resources. Yabi allows a scientist to submit, run, and monitor jobs from multiple operating systems and both desktop and mobile environments. It provides a domain-agnostic environment with a comprehensive suite of compute and data access models utilising SSH, GridFTP, Torque [21], PBS Pro [22], SFTP, S3 and others. This enables the Yabi system to leverage compute and data resources beyond those that are available on a local server or network. Yabi separates the roles of user, system administrator and software developer. This allows a system administrator to add tools to the environment using the Yabi administration web application without the need to undertake software development (see Section 3.1).

### 2 Yabi frontend application

User interaction with Yabi is via a web-based application written in Python and built around the Django Framework (https://www.djangoproject.com/). From a user's perspective the Yabi frontend application provides methods for creating, naming, submitting and monitoring workflows, and for accessing data on external storage systems. The Yabi frontend is designed to provide a REST [23] style web service to allow different client interfaces to interact with it. In the current implementation two user interfaces have been built: a web-based client and a command line client.

#### 2.1 Web-based client

**Figure 2:**
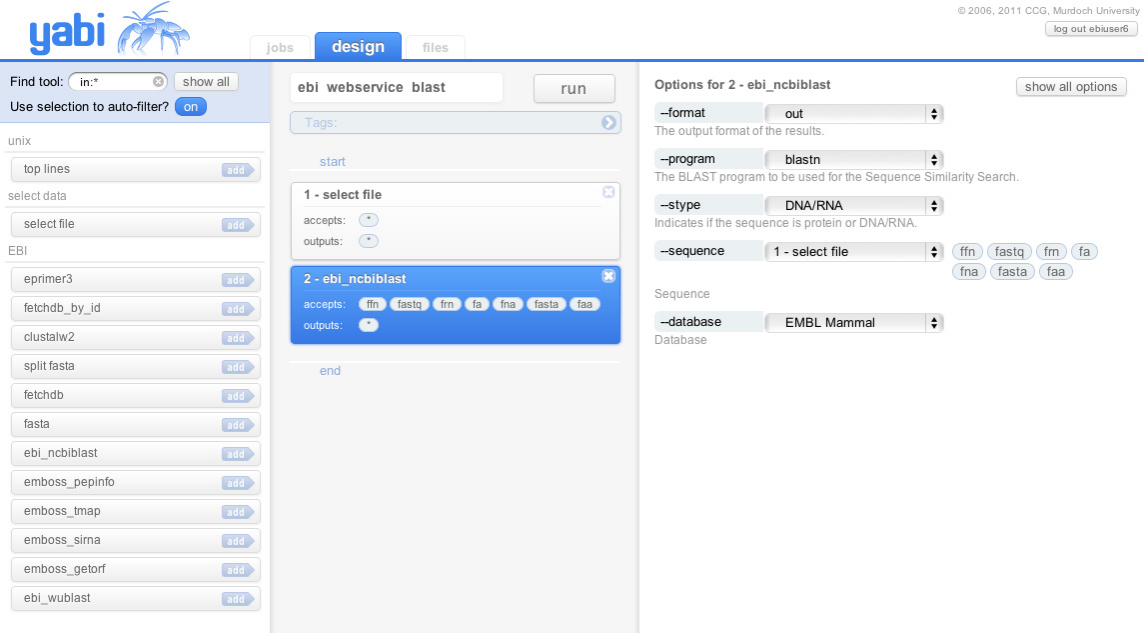
**Yabi web-based client**. Screenshot of the Yabi web-based client in the "design view" accessing a tool available from the European Bioinformatics Institute via a webservice (http://www.ebi.ac.uk/Tools/webservices/).

Typically, users would interact with Yabi through the Web-based client by establishing a session through an initial log in and authentication. The user is then presented with an interface consisting of three views: the design view, the jobs view and the files view, shown in Figure 2. In this example, Yabi is configured to invoke a simple sequence similarity search using web services running on remote services, in this case, the European Bioinformatics Institute (http://www.ebi.ac.uk/Tool/webservices).

The design view allows a user to construct a workflow by selecting each tool they wish to use and completing the tool's mandatory and optional inputs. The workflow may then be optionally named before it is submitted for execution. All workflows are stored as part of a complete audit trail and are available to be reused. Additional workflows may be run concurrently. Included in this design view are enhancements to the workflow construction such as automatic filters to highlight appropriate tools at a particular stage and providing visual cues to users of potential dependencies that cannot be resolved.

Previously submitted workflows are accessed via a jobs view. Comprehensive provenance for each workflow is kept including input files, all tools and each tool's parameters. Searching by name, date, and arbitrary metadata is also available.

Resulting files produced by workflows may be viewed either directly through the workflow representation in the jobs view, or via a files view which is a representation of the external data resources accessible by the user allowing typical file management tasks such as copying, deleting and renaming.

The design, jobs and files views provide drag-and-drop interfaces that abstract away the complexities of process management and interfacing with HPC environments.

Technically, to present the interface to the user, the client browser makes Ajax (http://www.adaptivepath.com/ideas/essays/archives/000385.php, 2011) calls to the frontend application. When information about tools and the state of workflows is required the frontend establishes a secure, authenticated connection to the middleware and retrieves the relevant information. For example, to display a list of tools for a user, an Ajax call is made from the web browser to the frontend application. The frontend application in turn makes a request to the middleware. The middleware then returns a JavaScript Object Notation (JSON) (http://tools.ietf.org/html/rfc4627, Oct 2011) list of tools, which is returned by the frontend to the client interface. An important point to note is the distinction drawn between each of the components. The HTML/JavaScript interface only communicates with the frontend application. The frontend then makes all connections with the middleware. Similarly, if the web client requires a file listing, a request is sent via the frontend to the middleware, which then requests a listing from the Resource Manager.

#### 2.2 Command line client

For users that prefer command line access, a command line client for Yabi, Yabish is provided. Yabish communicates with the frontend application to authenticate and establish a session. Once this is done it is possible for users to construct and submit jobs to Yabi via the command line. In addition, Yabish includes commands to list running jobs. Using Yabish enables users familiar with scripting languages to incorporate Yabi into their work environment or legacy systems. Results may then be fetched using Yabish or alternatively by logging into the web-based client and viewing them in a web browser. This enables groups of researchers with varying computing skills to share common tools. In this way, external workflow environments that have naïve execution and data access models can also be configured to utilise Yabish tools and then have access to a sophisticated execution and data access environment.

### 3 Yabi middleware

The second layer of the Yabi three-tier architecture is the Yabi middleware. The middleware consists of two modules; the first is referred to as "Yabi Administration" and is a web-enabled application that handles the description of all tools, users, data services and compute services; the second is referred to as the "Yabi Engine" and is an event driven system tasked with accepting and processing workflow requests.

#### 3.1 Yabi administration

The Yabi administration module allows a system administrator to manage all aspects of the Yabi system using a web browser. Tools are added by creating a tool record and filling in all necessary details. This includes the name of the program to be run, the compute resource that will perform the task, and where resulting files should be stored. Further to this, the system allows full specification of all parameters and file types used by a tool. Later, the Yabi Resource Manager uses these details when it executes a task and the frontend uses these details when it presents a visual representation of the tool to users.

**Figure 3:**
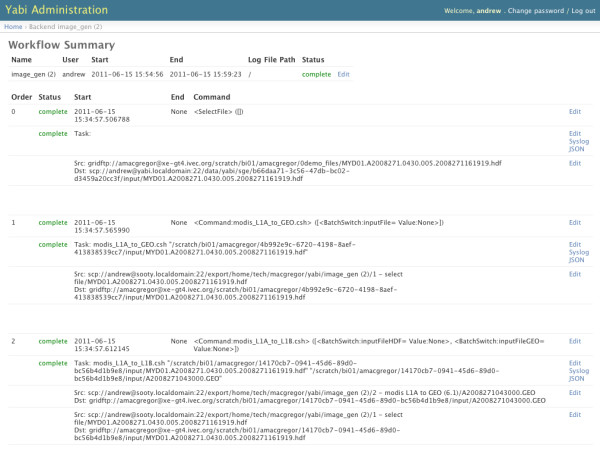
**Yabi Admin**. Screenshot of a completed workflow from a system administration perspective.

The Yabi administration also controls user access to compute and data services. It maintains records of users and optionally caches their credentials to access compute and data services. When the Yabi frontend requests a file listing for a particular data service a request is made to the Resource Manager with the correct credentials to retrieve that listing. Similarly, when a task needs to be run on a compute resource, a task is served to the Yabi Resource Manager along with the correct credentials. Figure 3 shows a section of the implemented Yabi administration interface. Specifically, this figure shows a summary of a running workflow including: i) workflow details including name, user and start and end times; ii) jobs that will be executed with a description of each command; iii) individual tasks with the actual command that will be executed and the files that will be staged in; iv) status of the workflow and each job and task; and v) a "Syslog" link for each task to view the system logging received from the Yabi Resource Manager.

#### 3.2 Yabi engine

The second module of the Yabi middleware appliance is the "Yabi Engine". This module is responsible for accepting and processing workflow requests originating from the Yabi frontend, creating descriptions of the processing required for the individual workflow steps ("jobs") and submitting them to the Yabi Resource Manager for execution on local or external compute resources. External resources include existing HPC, Grid and Cloud.

When the user creates or chooses to reuse a workflow, and then executes it, the Yabi frontend submits a high level description of the entire workflow to the workflow engine. This description includes the following information for each step of the workflow: the tool, the parameters of that tool and their settings, the related input and output filenames for individual files and descriptions of any dependent file input requirements. This allows the Yabi engine to resolve any file dependencies dynamically as a workflow executes. For example, a particular tool may require the output from a previous tool in a workflow. The description for the latter tool will contain a pointer specifying that it requires the output from a previous tool, even though the specific files output from that tool are not yet known.

The Yabi engine processes incoming workflows in a separate thread using an external message queue. The benefit of this strategy is that it allows a timely response to the Yabi frontend by detaching the processing of incoming workflows from the frontend request. Once processed the workflow is stored in an SQL database prior to the workflow being executed.

At each step through the workflow execution, the Yabi Engine examines the workflow, resolving dependencies. If all the dependencies for a job are resolved, that is, all the required input files are present and all the parent jobs have finished executing, then the required execution tasks are created for the job. Each task represents an execution process on an execution backend that is associated with the tool being used in that job. When all the tasks of a job are finished, the next step in the workflow is processed. Processing of the workflow continues until all the jobs in the workflow are complete or in an error state, at which point the workflow is marked as complete, or with an error flag respectively.

To determine whether a job's dependencies have been satisfied, the Yabi Engine utilises the concept of "file types". One or more of a tool's input parameters may be marked as accepting a certain file type. These file types are specified in the database as a unix-style filename matching pattern. Any files that match the pattern for the tool's input parameter are able to satisfy the requirements of that input parameter. For instance, if in the workflow there is a job that requires the output of a previous step in the workflow, then any appropriate files from that previous step are used to generate the tasks for this job.

If a tool has multiple input parameters that take different file types, and these parameters are both specified as coming from previous jobs in the workflow, then once the previous jobs are complete their corresponding output files are grouped together to create a batch of tasks for that job. To match up an input file of one type for one parameter with the correct input file of another type for another parameter, then the files with minimum Levenshtein distance [24] are grouped together to create each task.

### 4 Yabi resource manager

The third layer of the YABI three-tier architecture is the YABI Resource Manager. The Yabi Resource Manager handles all the tasks involved in using different network protocols to access compute resources and in the streaming of data between different data resources. The Yabi Resource Manager can perform any execution submission task on any execution backend. Likewise it can perform any file system operation on, or between, any file system backend. The Yabi Resource Manager takes commands and credentials from the Yabi Middleware and does so over a secure authenticated connection.

The Resource Manager is not a job scheduler, scheduling is handled by each external compute resource. The various aspects of the Yabi Resource Manager are outlined in the following sections.

#### 4.1 Design

The Yabi Resource Manager is programmed in Python (http://www.python.org/) and makes use of gevent (http://www.gevent.org/) and greenlet (http://pypi.python.org/pypi/greenlet) for more advanced features like co-routines [25] and micro-threads. It also uses the Twisted framework (http://twistedmatrix.com/), an event driven network engine.

The structure of the Yabi Resource Manager is based around the Twisted Python approach of using a small number of threads (typically one per CPU core) but handling a large number of network connections simultaneously. When a connection is made, existing threads handle the connection rather than starting a new thread, as is the case in typical threaded server architecture. Each thread manages its time in an event-driven manner while handling all the connections allocated to it. This has the advantage of being able to handle an extremely large number of connections simultaneously.

The Yabi Resource Manager provides two types of services that we will discuss: data services and compute services (Section 4.4).

#### 4.2 Data services

This service has a plug-in architecture that allows it to talk to a variety of different file storage systems. It uses a Uniform Resource Identifier (URI) (http://www.w3.org/TR/uri-clarification/) style path descriptor to define file system locations and the protocols the resource manager should employ to access them.

The data services exposed by the Yabi Resource Manager are: directory listings; file copying (between the same or different data resources); file deletions; and directory creation. These are exposed as web service calls with the requested files or directories being passed in as HTTP parameters in the form of a URI. An example of such a URI is ssh://username@hostname.domain/path/to/file. By specifying different schema in the URI, the Resource Manager can utilise different protocols to transfer the data or perform the operation.

The Yabi Resource Manager performs file system operations by spawning helper processes that perform the task and keep a handle on its standard out and standard error streams. By using these streams in conjunction with the exit code of the process, the Yabi Resource Manager can determine if a task succeeded or failed and the reasons for this. For copy operations the system is designed to always stream the data and no data is written to disk when copying data from one data service to another.

Every copy task is comprised of a subtask that writes to a FIFO (http://www.kernel.org/doc/man-pages/online/pages/man7/fifo.7.html), and a subtask that reads from the same FIFO. A FIFO is a special file that may be accessed as part of the file system. When passed data using a FIFO, the operating system kernel handles it internally in memory rather than writing it to disk. In this way the transfer of the data is performed by the underlying operating system in a very efficient manner. As long as a file system service has a command line tool or application programming interface (API) then it can be integrated into the Yabi Resource Manager.

#### 4.3 Credentials

Yabi has a comprehensive model for managing the credentials required to access external compute and data environments. The credential model allows the requirements of two competing use cases to be satisfied. Firstly, users can choose to let the application securely store encrypted credentials on their behalf or secondly users can exercise fine grained control of the storage and expiry of credentials from the system.

Credentials may be stored, encrypted, in the database to be utilised by the application as required. In this case, the credential is encrypted with the Advanced Encryption Standard (AES) using the user's password to generate an encryption key. When a user logs into the frontend, the encrypted credentials for the user are taken from the database and decrypted using their password based key. Once decrypted the credentials are cached in RAM for use by the system.

In the case where the system asks for a credential that is not available in a decrypted form, the running job is placed into a blocking state so that the user can resume the job once they provide the necessary credential.

#### 4.4 Compute services

The compute service uses a plug-in architecture to submit jobs to a variety of different execution resources. The Yabi Resource Manager periodically connects to a web service exposed by the Yabi middleware application and requests any tasks that are ready for execution. The Yabi middleware returns a snippet of JSON that describes the task including URIs for the Yabi Resource Manager to call to report status and log messages. The Yabi Resource Manager then handles each task as a greenlet tasklet. When executing tasks the Yabi Resource Manager performs all necessary operations such as creating relevant directories, staging in and out the requested data, executing the remote task, monitoring the task until it is finished and cleaning up temporary files.

As it does this, the Yabi Resource Manager returns any task status changes to the Yabi middleware, which can in turn provide these to the Yabi frontend for display to the user. The Yabi Resource Manager also allows for task resumption, a feature that assists in stopping and starting the application.

#### 4.5 Shutdown and startup

It is essential that the Yabi Resource Manager can be restarted without affecting any running jobs. For this reason the Yabi Resource Manager serialises all tasklets to disk during shutdown. When it starts up again it deserialises the tasklets from disk and attempts to resume them. Any stale connections that fail will be retried by the tasklet. If this fails the tasklet will attempt to continue the task by retrying the last action.

#### 4.6 Fault tolerance & error handling

To facilitate fault tolerance and error handling of external transient events, such as credential errors, file permission errors or unavailability of execution resources, there is a subset of exceptions in Yabi that are descended from a special "BlockingException" object. This exception reports a "blocked" rather than an "error" state in running tasklets. In this way, temporary failures that need user or administrator intervention can return this blocking exception. Tasks that enter a blocked state can be resumed once the temporary failure has been addressed rather than failing permanently.

### 5 Summary features of yabi architecture

The design of the system lends Yabi to have a number of desirable features, summarized by: i) Internet-based application; ii) provides transparent HPC access and flexible across multiple protocols (Eg. GridFTP, Globus, SSH via pluggable backends); iii) able to link analysis tools together across resources; iv) error handling (e.g. retry, block, fail and report); v) web-based comprehensive administration of tools; vi) abstract complexities from users; vii) can incorporate any command line analysis tool (E.g. Java, Perl, Python, C, R, etc.); viii) credential handling; ix) simplified access to web services; x) batch processing; and xi) command line access of Yabi tools.

The features of the Yabi user interface can be summarized by: i) ease of use; ii) design/reuse workflows; iii) audit trails/provenance; iv) data/results not locked into system (results stored on existing file storage resources, and not within Yabi itself); v) mix and match tools from multiple execution environments in any given workflow; vi) analysis tools integrated can either be open source or proprietary; vii) meta data capture of workflows and search (available analysis tools or previous workflows); and viii) file management between disparate storage resources.

## Results and discussion

**Figure 4:**
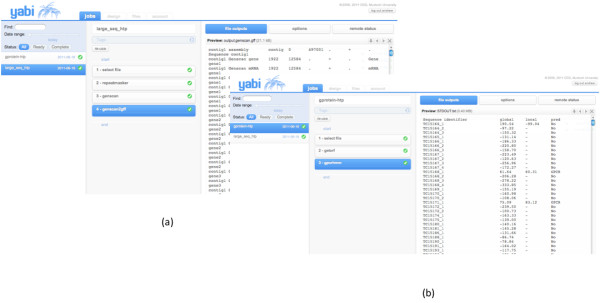
**HTP Genomic and Automated annotation workflows**. Screenshots of (a) a high throughput genomic analysis workflow; and (b) a bioinformatics workflow to predict candidate G-Protein coupling receptor proteins batched over 14,000 molecular sequences.

The power and flexibility of Yabi is outlined by presenting three typical use cases. In the first use case [26] analysis for high throughput genomic analysis is conducted on a relatively large molecular sequence (approximately 500 kbp in length). In Step 1 (select file) is used to select the input file. In Step 2 an analysis tool (repeatmasker - http://www.repeatmasker.org/) is used to identify and "mask out" the known repetitive molecular sequence elements from the sequence. In step 3, a prediction tool (genscan [27]) is used to predict candidate genes within this sequence and step 4 (genscan2gff [4]) formats the output from Step 3 into standard GFF (http://www.sanger.ac.uk/resources/software/gff/spec.html) file format for uploading into genome browser visualization tool. The screenshot of the workflow is shown in Figure 4a.

Figures 4a and 4b show the execution status of example workflows using the jobs view. Additional workflows can be run concurrently by returning to the design view. The jobs view also provides access to previously completed workflows. Comprehensive details of each workflow are kept in an audit trail database including all tools, parameters and input files used. A files view provides direct access to the underlying file system for each user. Other workflow examples include: sequence assembly, large scale similarity searches and sequence manipulation.

In the second use case [26], the workflow analyses a large number of DNA molecular sequences (14,000 sequences) and conducts a predictive analysis for candidate G-Protein coupling receptor proteins, shown in Figure 4b.

In step 1 (select file) a file is selected containing the 14,000 sequences. The file can be selected from any file system resource made available by the administrator to this user. In this example the file system selected is attached to the local HPC. Step 2 (getorf [28]) predicts the translations of each of the 14,000 sequences to the equivalent amino acid sequences and finally in step 3 (gpcrhmm [29]) candidate G-Protein coupling receptor proteins are predicted.

**Figure 5:**
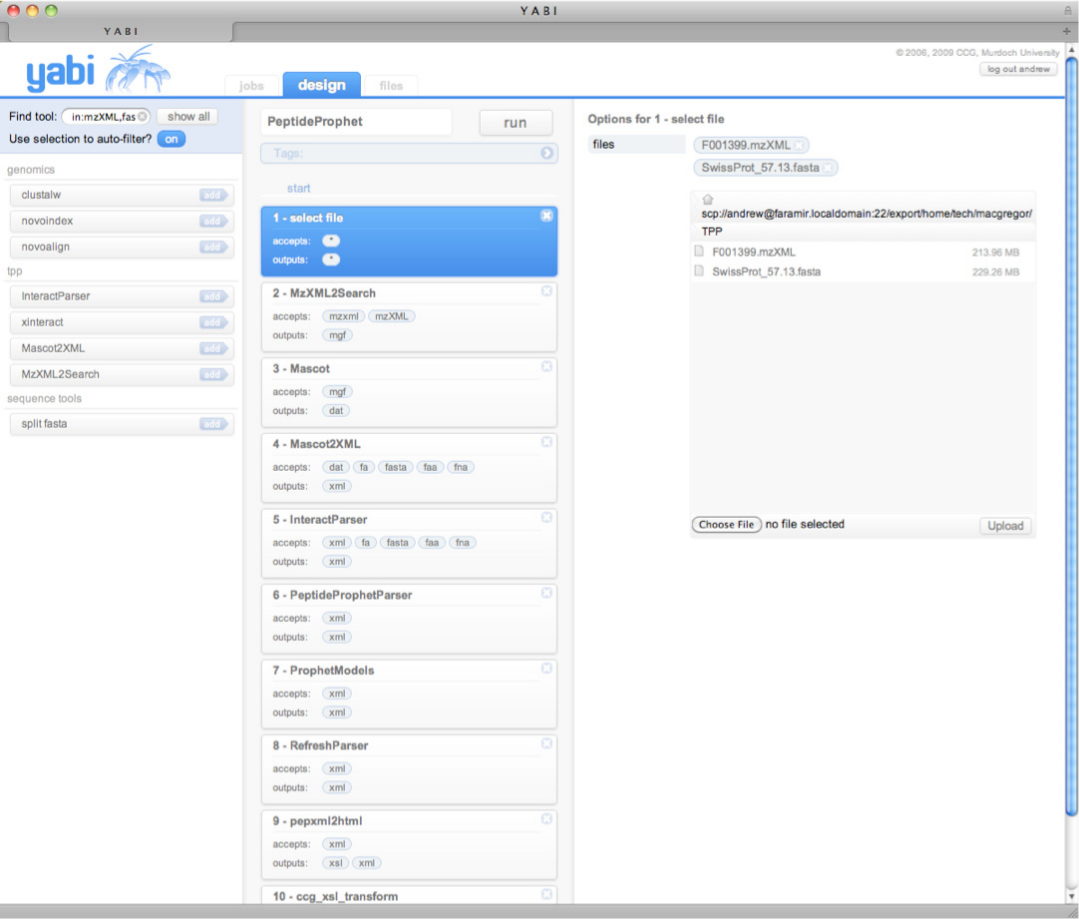
**Proteomics Analysis Workflow**. Screenshot of Proteomics workflow combining tools from TPP and Mascot.

In the third use case, core Trans-Proteomic Pipeline TPP tools [30] have been made accessible to enable user level workflow creation within Yabi rather than using the inbuilt web interface Petunia which can only be customized by systems administrators. The majority of tools contained within TPP can run on a command line which means that incorporation into the YABI environment is possible. Having tools within Yabi enables researchers to use different search engines or quantitative tools that might not be available in TPP or accessed through the Petunia web interface. To demonstrate this feature, Figure 5 shows a screenshot of a typical proteomics workflow within Yabi linking tools from TPP (MzXML2Search, Peptide Prophet [31]), as well as the Mascot [32] search engine.

Within each of these use cases, most of the tools selected as part of the workflows are computationally intensive and require running on HPC infrastructure. However, there are other tools/scripts that can be configured to run on a standalone Linux machine (for example genscan2gff). These examples demonstrate that the Yabi environment is able to "mix and match" tools configured on HPC resources and regular servers and made available transparently to users. Yabi has also been recently used in the analysis of metagenome data [33] (figure not shown).

## Conclusions

The main aim of the Yabi project is to provide transparent access to existing HPC resources. This has been achieved by the development of a three tier architecture that separates the roles of end user, system administrator and software developer. Yabi has a plug-in architecture that allows the system to interface to a variety of different storage and compute systems. This architecture prevents data lock-in as Yabi facilitates seamless access to existing storage resources. Yabi is currently in production use for a number of research communities in the life sciences in genomics, transcriptomics and proteomics. Yabi is complementary to existing workflow environments as it targets the non-technical audience with a rich and intuitive web-based user interface that allows the uptake of HPC by a broader audience. Yabi enables other workflow systems, that possess limited functionality to access multiple existing HPC systems, to leverage Yabi's data and access models via its command line client. The system is designed to be domain-agnostic which means Yabi can service many life science domains as well as enable cross-disciplinary research such as systems biology and marine science. The Yabi project is open source and can be downloaded or accessed at the project website http://ccg.murdoch.edu.au/yabi.

## Availability and requirements

**Project name: **Yabi

**Project home page: **http://ccg.murdoch.edu.au/yabi/

**Operating system(s): **Linux

**Programming language: **Python

**Other requirements: **memcached, sqlite

**License: **GNU GPL v3

**Any restrictions to use by non-academics: **No

## Competing interests

The authors declare that they have no competing interests.

## Authors' contributions

Original concept and design: MB and AH; Architecture: AH; Lead design: AH, CW, AM; Implementation: AH, CW, AM, TS; Wrote manuscript: MB, AH, AM, CW. All authors read and approved the final manuscript.

## Authors' information

**Adam Hunter**: Adam Hunter completed a BSc Honours in Computer Science at Murdoch University. Adam has over 10 years experience in ICT including software development in C and Java. He leads the CCG software development and infrastructure team. Current areas of focus include continuous integration, agile programming and high performance and cloud computing.

**Andrew Macgregor**: Andrew Macgregor has a BA in English from the University of Otago and has also studied Computer Science at Massey University. He is a software developer focusing on Internet Application development using Python. Previously, Andrew contributed the UniGene module to the Bioperl project.

**Tamas Szabo**: Tamas Szabo has a Bachelor of Science in Computer Engineering, Gabor Dennis College, Budapest. He is a software developer who has worked in Australia and overseas in many sectors including banking, health, entertainment, government, travel, mining and research. Tamas has an interest in programming languages, development methodologies, open source development, operating systems and networks.

**Crispin Wellington**: Crispin Wellington completed a BSc in Physics at Curtin University. He is a software engineer who utilises Python and he also has an interest in functional languages such as Lisp. Crispin is interested in the human factors that affect the development of architecturally consistent systems.

**Matthew Bellgard**: Professor Matthew Bellgard completed a BSc Honours and PhD in Computer Science from The University of Western Australia. He is Murdoch University's Bioinformatics Chair and the Director of the Western Australian State Government Centre of Excellence, the Centre for Comparative Genomics. His scientific work has resulted in developments in the areas of pairwise sequence alignment and artificial intelligence, early detection of base composition differences in closely related bacterial species, whole genome sequence analysis and advances in the development of web-based integrated systems utilising high performance computing.
